# Textural and Rheological Properties of Sliceable Ketchup

**DOI:** 10.3390/gels9030222

**Published:** 2023-03-14

**Authors:** Nadia Shokraneh, Mazdak Alimi, Seyed-Ahmad Shahidi, Maryam Mizani, M. Bameni Moghadam, Ali Rafe

**Affiliations:** 1 Department of Food Science and Technology, Ayatollah Amoli Branch, Islamic Azad University, Amol P.O. Box 6616935391, Iran; 2 Department of Food Science and Technology, Science and Research Branch, Islamic Azad University, Tehran P.O. Box 6616935391, Iran; 3 Department of Statistics, Allameh Tabataba’i University, Tehran P.O. Box 1489684511, Iran; 4 Department of Food Processing, Research Institute of Food Science and Technology (RIFST), Mashhad P.O. Box 91775-1163, Iran

**Keywords:** sliceable ketchup, gum, viscoelastic, rheological properties, syneresis

## Abstract

This study investigates the effect of different mixtures of gums [xanthan (Xa), konjac mannan (KM), gellan, and locust bean gum (LBG)] on the physical, rheological (steady and unsteady), and textural properties of sliceable ketchup. Each gum had an individually significant effect (*p* < 0.05) on viscosity; however, the addition of Xa in combination with other gums had a greater effect on viscosity. By increasing the use of Xa in ketchup formulations, the amount of syneresis decreased such that the lowest amount of syneresis related to the sample prepared with 50% Xa and 50% gellan. Although the use of different levels of gums did not have a significant effect on the brightness (L) and redness (a) indices (*p* < 0.05), the use of different ratios of gums had a significant effect (*p* < 0.05) on the yellowness (b) index. The effect of different levels of gums used had a significant effect only on firmness (*p* < 0.05), and their effects on other textural parameters were not statistically significant (*p* > 0.05). The ketchup samples produced had a shear-thinning behavior, and the Carreau model was the best model to describe the flow behavior. Based on unsteady rheology, G’ was higher than G” for all samples, and no crossover between G’ and G” was observed for any of the samples. The constant shear viscosity (η) was lower than the complex viscosity (η*), which showed the weak gel structure. The particle size distribution of the tested samples indicated the monodispersed distribution. Scanning electron microscopy confirmed the viscoelastic properties and particle size distribution.

## 1. Introduction

Currently, market research shows a growing trend in ketchup consumption [1]. It is predicted that in the period from 2021 to 2025, the market for this type of product will have an annual growth of about 2.64%. Ketchup is a flavored product containing various ingredients produced from a combination of fresh tomatoes or concentrates in the form of puree or pastes with sweeteners, salt, vinegar, and spices [2]. This type of sauce is a non-Newtonian semisolid material with a yield stress that exhibits thixotropic and viscoelastic properties [3,4,5,6].

Ketchup is consumed with various foods such as ready meals, pasta, pizza, etc. One of the most widely used uses for ketchup is with meat products, such as sausages, and since these two types of foods are commonly consumed together, their combined use can be a unique and attractive topic. Currently, a mixture of hydrocolloids is used to improve physical and rheological properties and reduce costs [7]. Commercial ketchup usually is thickened with tomato pulp powder [7], potato or corn starch [8,9], modified starch and carboxymethylcellulose [10], guar, and carboxymethylcellulose and Xa [3,11].

Ketchup for use in combination with sausages, as a final product, must have a high viscosity, and in terms of texture properties, it should be elastic and solid-state, and if cold, it can be cut and is sliceable like a sausage. Binary composite hydrogels of alginate and guar gum have been applied in the preparation of restructured pimento strips [12,13]. Therefore, due to their different physical and rheological nature from regular ketchup, the gel and sliceable texture of this type of ketchup are due to the application of gelling hydrocolloids such as Xa, gellan, KM, and LBG, especially considering their synergistic properties with each other [14].

Xanthan (Xa) gum shows high viscosity at low concentrations and has excellent stability in hot and acidic media [15]. The Xa has a highly pseudoplastic behavior and is synergistic with galactomannans (LBG and guar) and KM, increasing the viscosity of the solution. When Xa gum is mixed with KM gum or LBG, they form heat-reversible gels. The strongest gels in the presence of salt are 2:1 for the Xa–KM mixture and 1:1 for the Xa–LBG mixture [16,17]. Some researchers reported that in xanthan/konjac glucomannan mixtures, the maximum gel strength occurred at the ratio of xanthan to KGM = 4:6 without salt, and at 1:1 in the presence of sodium salt [18]. Renou et al. (2013) showed that a combination of Xa gum and locust bean gum showed strong rheological properties due to specific interactions between the two gums [19]. This follows the rubber elasticity theory for locust bean and Xa gums, as the increase in viscoelasticity due to heat treatment is related to the increase in crosslinking density between locust bean smooth areas and disordered regions of Xa gum [16]. Furthermore, a mixture of Xa gum and KM shows strong synergistic effects, and this effect is more seen in the ratio of 3:7 (KM:Xa) and offers the highest viscosity in all shear rates [20]. The KM gum also has an excellent synergistic effect as a strong gel with hydrocolloids, such as agar, Xa, and carrageenan, and creates heat-reversible gels. Various studies have reported the rheological properties and behaviors of solutions of this gum and its effects on other hydrocolloids [20,21,22,23]. Mirzaei et al. (2018) showed that using a mixture of KM and Xa gum in ketchup improves the physical stability and color of the sauce [24]. Gellan gum can also create a wide range of gel textures from hard and brittle gels to elastic, and as a multifunction gum with different functional properties, it is widely used in the food industry as a gelling, texturing, stabilizing, suspending, and structuring agent [24,25,26,27].

Various studies have been carried out on the synergistic interaction between some hydrocolloids in enhancing and improving the viscosity, texture, and functional properties of food [24,28,29]. However, there is a lack of knowledge on sliceable ketchup. Therefore, this study aimed to investigate the effect of gelling hydrocolloids on the physical, textural, and rheological properties of ketchup and to develop a new formulation for sliceable ketchup and its combined application as a filler in meat products such as sausages.

## 2. Results and Discussion

### 2.1. Viscosity

The viscosity of the ketchup is an essential factor in its acceptance by the consumer; therefore, it is also considered in the commercial production of ketchup [30,31]. Due to the unique nature of sliceable ketchup and its application in different sausages, this parameter was measured in the cooking temperature range and consumption of meat products (80–70 °C). According to Table 1, the addition of each of the gums (Xa, KM, gellan, and LBG) separately or in combination with Xa gum had a significant effect on the viscosity of the samples produced.

**Figure 1:**
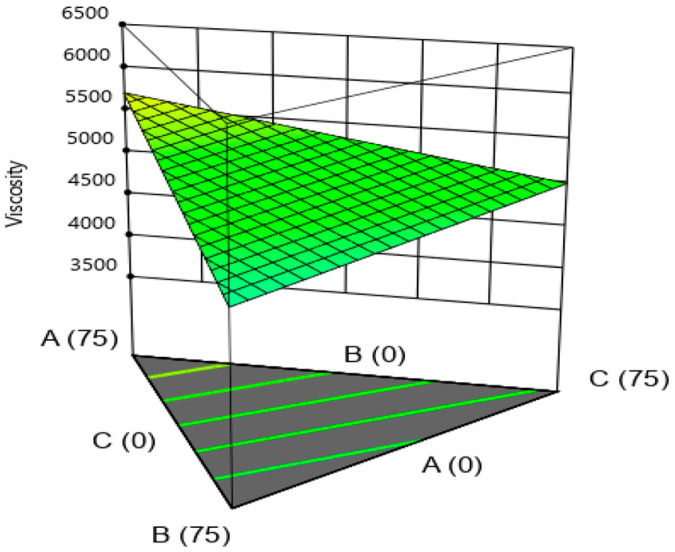
The effect of hydrocolloids on the viscosity of ketchup samples (A: xanthan, B: gellan, C: LBG).

As can be seen from Figure 1, during the combined application of hydrocolloids, the viscosity of ketchup samples decreased with increasing gellan content, while the viscosity of the produced samples increased with increasing amounts of Xa gum in combination with other hydrocolloids.

The lowest viscosity of the sample (12.5% Km, 12.5% LBG, 62.5% G, and 12.5% Xa) is K5, which could be due to the pseudoplastic behavior of the gel in the temperature range of 25 to 55 °C, and the Newtonian behavior of its solutions is in the temperature range of 70–60 °C. The gradual change in the behavior of the gellan solution from pseudoplastic to Newtonian, which is called gellan melting, is influenced by the re-formation of its structure as random macromolecular chains [32]. However, the presence of Xa in higher amounts increased the sauce’s viscosity, so that in the treatment, K13 (12.5% Km, 12.5% LBG, 12.5% G, and 62.5% Xa) shows the highest apparent viscosity among the samples. This increase occurred due to the synergistic effect between Xa gum, LBG, and KM.

Although it has been well established that the apparent viscosity of ketchup depends on the soluble pectin and pulp content relative to the total solids content [33,34], the effect of the concentration of hydrocolloids, especially Xa gum, on the viscosity is remarkable. In fact, trisaccharide units that are regularly located on the cellulose chain are responsible for the specific rheological behavior of this gum. Studies show that LBG exhibits high-viscosity non-Newtonian behavior alone [35,36]. However, in combination with Xa, due to intramolecular interactions between the Xa side chains and the main galactomannan chain, which follows the lock and key model, increase the viscosity of the sauce, which depends on the ratio of gums used in the formulation [35]. The interaction between LBG and Xa occurs due to the hydrogen bond between the LBG hemiacetal oxygen atom as the electron donor and the No. 2 carboxyl carbon group of the Xa side internal chain as the electron receptor [5]. An electrostatic bond also occurs between the K^+^ cation attached to the oxygen atom of the carbonyl group in the Xa glucoronosyl chain with the hemiacetal oxygen atom of the main LBG chain. The combination of Xa with KM and LBG also produces heat-reversible gels; a mixture of Xa and KM forms a stronger gel than the combination of Xa and LBG in equal percentages [35]. The glucose-free regions of the Km chain communicate with the smooth area of the Xa helixes, which in turn leads to an increase in viscosity.

Mao et al. (2012) described the synergistic effect between KM and Xa as the result of successive linkages between Km glucose sequences with chains of six units or more of Xa [37]. They believe that the bond between Xa–Km depends on the sequence of glucose units in the chemical structure of both gums as well as the high ratio of the enthalpy to the entropy of their reaction. As a result of intramolecular reactions between KM and Xa, dehydration occurs and water molecules are released, which causes a large change in their hydration entropy [35]. In addition, interactions between thickening molecules have been reported to increase viscosity [31]. In high-viscosity systems, the molecules involved in the gel lattice are more closely intertwined and the dispersed phase motility is low. Therefore, in the gel networks formed in the presence of KM, LBG, and Xa, especially in the K13 sample, the movement of the continuous aqueous phase is restricted in the ketchup suspension.

### 2.2. Physical Stability

The separation of ketchup serum (syneresis) is one of the biggest problems in the ketchup industry. Its control during storage is very important because it has a negative impact on product quality and acceptance by the customer [35]. Since this particular type of sauce will be used in meat products, high physical stability and low syneresis are very important. The results relating to the physical stability of different samples are shown in Table 2. As can be seen, the lowest amount of syneresis or the highest physical stability relates to the samples K6 (50% gellan, 50% Xa), K14 (100% Xa), K16 (50% Xa, 50% KM), and K7 (50% Xa and 50% LBG).

**Figure 2:**
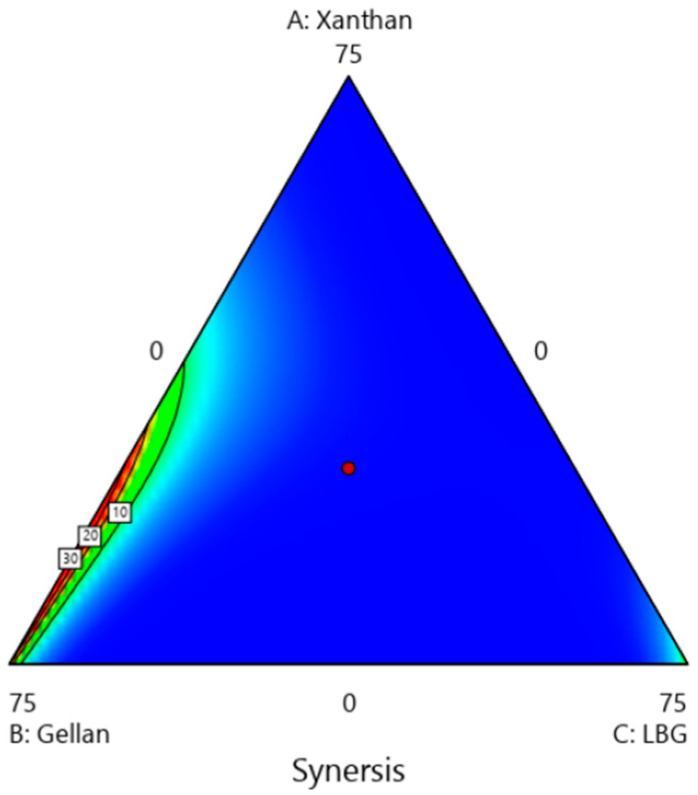
The effect of hydrocolloids on the syneresis of ketchup samples (A: xanthan, B: gellan, C: LBG).

In general, samples with higher viscosity also had better physical stability. The greatest effect on reducing syneresis was observed in the combined use of Xa gum and gellan. The mere use of KM and LBG alone has increased syneresis; however, when used in combination with Xa gum, they prevented syneresis of the samples. According to Figure 2, it can be seen that in higher amounts of Xa, the percentage of syneresis decreases and reaches below 1%, while increasing the amount of KM and gellan increases the rate of syneresis.

The high water-binding capacity of Xa gum (232 mL/g) [38] makes it very stable against syneresis even during storage because Xa gum is a heteropolysaccharide that has repeating pentasaccharide units. This pentasaccharide contains two units of mannose, two units of glucose, and one unit of glucuronic acid; therefore, these factors cause the stability and firmness of Xa gum against acid and heat. Another reason is the synergistic effect between Xa and gellan [39]. When Xa and gellan are used, the amount of syneresis is reduced to an acceptable level. This positive effect is enhanced when a 1:1 ratio of these two gums is used in the formulation [40]. For this reason, in the confectionery industry, the combination of gellan with Xa and LBG is used to improve and maintain moisture and reduce the syneresis of a variety of jelly desserts and puddings [16].

### 2.3. Color Properties

The L* index indicates the product’s lightness and is in the range of zero (completely dark) to 100 (completely light). The parameters a and b are in the range of −60 to +60. Positive values of the a parameter indicate redness, and negative values indicate the amount of green. Further, the b parameter indicates the amount of yellowness and blue in the sample. The results of the colorimetric test of the samples are shown in Table 3.

**Figure 3:**
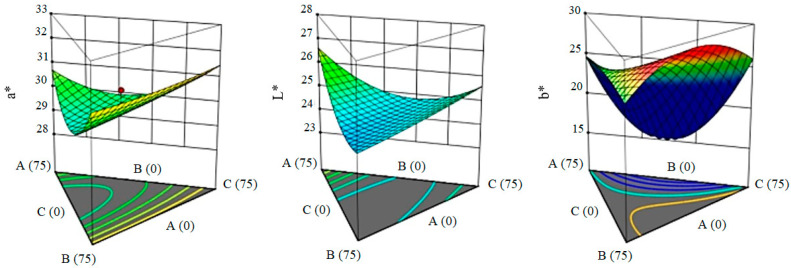
The effect of hydrocolloids on the color of ketchup samples (A: xanthan, B: gellan, C: LBG).

According to Figure 3, the effect of applied hydrocolloids on the color parameters L, a, and b is observed. Since the total concentration of gums used in all samples is equal (in total: 1.5%), their different concentrations have no significant effect on the parameters L and a and are effective only on the b parameter. The concentration of Xa and gellan in the formulation of this index has also increased. The highest value of the a is for sample K8 (50% Xa, 50% G), and the highest L is for sample K14 (100% Xa).

The a/b ratio is often used as a qualitative parameter in tomato-based products [41]. This ratio indicates that the finer the particles, the more and easier it will be to detect their lycopene content. Therefore, in this case, the ratio a/b will be more than one [8,42]. The results obtained for the parameters a and a/b were consistent with the results of Poretta (1991) on 18 types of commercial ketchup [4].

### 2.4. Texture Analysis

The results relating to the textural characteristics of different ketchup samples can be seen in Table 1. The evaluation of various parameters of cohesiveness, springiness, gumminess, chewiness, firmness, and adhesiveness showed that process variables only have a significant effect on texture firmness. Except for the samples containing Xa gum and LBG in other samples, the addition of each gum alone or in combination did not have a significant effect on the firmness. The K13 and K19 samples had the highest firmness

**Figure 4:**
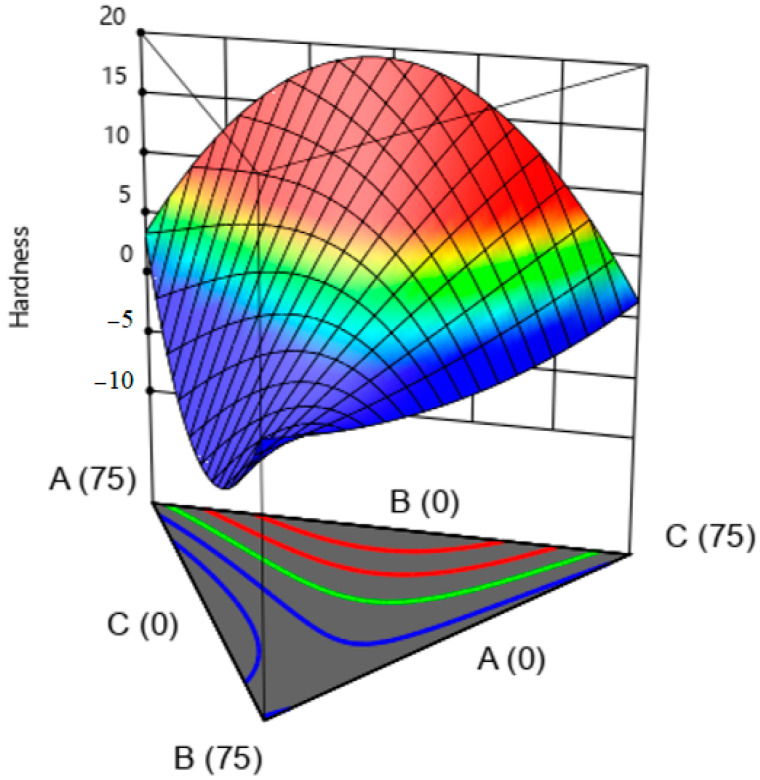
The effect of hydrocolloids on the hardness of ketchup samples (A: xanthan, B: gellan, C: LBG).

According to Figure 4, it can be seen that with higher amounts of Xa and LBG, the firmness of the ketchup sample increased, while on the other hand, by increasing the amount of gellan, the firmness decreased. This is also due to the synergistic effect between Xa gum and LBG because Xa gum does not form a gel on its own. The texture firmness results, for example, K14, which contains 100% Xa (Table 4), confirm this. However, the combination of these two gums with each other due to the synergistic effect and intermolecular interactions has led to increased firmness; the results of Tako (1992) confirm this [43].

According to the results of viscosity, physical stability, color, and texture analysis, two samples of ketchup with optimal formulas along with a sample of commercial ketchup as a control sample were selected for flow behavior tests, frequency sweep, particle size distribution analysis, and microstructure analysis.

### 2.5. Rheological Analysis

This test was performed on two selected samples and a sample of commercial ketchup as a control. The rheological properties of the samples were evaluated in two sections: flow behavior and viscoelastic properties. These tests were measured for the commercial sample of ketchup at 25 °C and the optimal samples at 55 °C. The temperature of 55 °C was also selected according to the sausage temperature upon consumption.

#### 2.5.1. Flow Behavior

**Figure 5:**
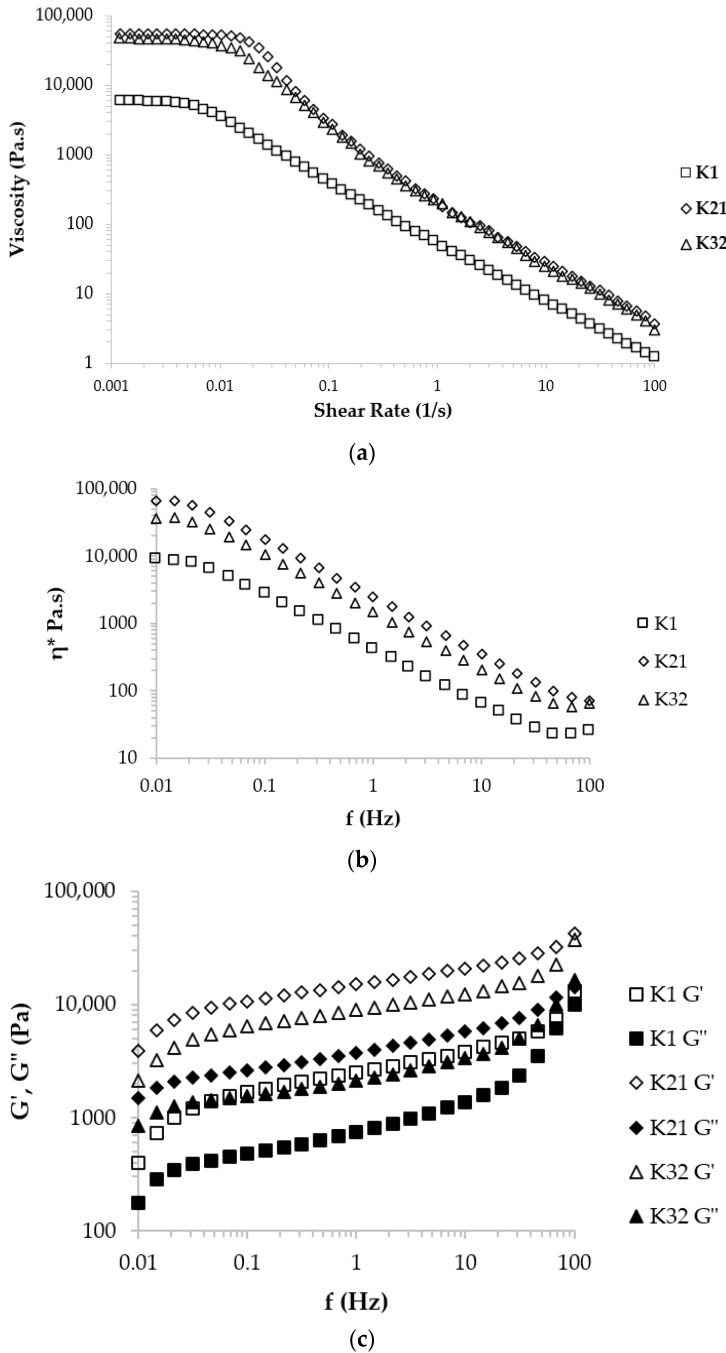
Rheological properties of ketchups (commercial and sliceable): (**a**) flow behavior, (**b**) complex viscosity, and (**c**) storage and loss moduli as a function of frequency. (K_1_ = 100% xanthan and 0% LBG, K_21_ = 24.939% LBG, and 75.061% xanthan, K_32_ = 67.906% LBG, and 32.094% xanthan).

The apparent viscosity trend changes in the selected samples against frequency are shown in Figure 5a. The results show that the viscosity of all samples decreased by increasing the shear rate, which indicates the pseudoplastic and shear-thinning behavior. The commercial sample (K1) has a much lower viscosity than the other two samples (K21 and K23). Similar flow behavior has been reported in various studies for ketchup [3,5,44,45,46,47,48]. In fact, due to the synergistic interactions between Xa and LBG, the functional groups of the two gums participate in the water uptake process and form a gel network.

Due to the increase in shear rate and the alignment of the biopolymer chains of hydrocolloids in the gel network with the applied incision, the gel structure is degraded and consequently, the apparent viscosity decreases and the shear-thinning behavior appears [45].

For galactomannans and other polysaccharides with a random helix structure at very low and very high shear rates, Newtonian behaviors called zero and infinite viscosity have been observed [49]. Rheological models such as Cross and Carreau are used to evaluate this behavior. In this study, the data were fitted with different rheological models and the results of fitting showed that the Carreau model with the highest coefficient of explanation (R^2^) is a suitable model to describe the results of the flow behavior of the samples. Table 5 shows the various parameters of the Carreau model.

Samples K21 and K32 showed the highest infinite viscosity. A comparison of zero and infinite shear viscosities (η_0_ and η_∞_) shows that adding Xa and LBG in these ratios reduces the shear viscosity. This can be attributed to the interaction between the two gums, which is also consistent with published results [45,46]. The rest time (λ) was positive for all samples, indicating non-Newtonian behavior.

At low frequencies in all three samples, the phenomenon of zero shear viscosity was observed, which indicates the ability of the samples to be two-phase at low frequencies. In this case, the K21 sample (with a viscosity of 8890 Pa·s) is better because it has a higher viscosity than other samples, especially the commercial sample. This is due to the LBG and Xa in the two samples K21 and K32 because the synergistic effect between these two gums had reached its maximum value. This confirmed the interactions between the free LBG galactose groups and the Xa chain, which led to the formation of a strong gel network [50]. Additionally, at higher frequencies, there is a significant difference between the viscosity of the commercial sample and other samples. Due to the use of this type of sauce as a filling in sausages, this parameter is essential.

Since the value of n samples (Table 2) is in the range of 0.54–0.45 and all are less than one, it indicates the shear-thinning (pseudoplastic) behavior due to shear force for all samples. Sharoba et al. (2005) reported the flow behavior index of commercial ketchup samples from 0.399 to 0.275 [2].

Regarding the changes in the complex viscosity with the frequency, the viscosity in all three samples reaches a constant limit at very low frequencies, which indicates the Newtonian behavior of the samples in the static state. Then, by increasing the frequency and applying the stress, the viscosity decreases and shear-thinning behavior is observed in all samples. The high viscosity of the K21 sample is due to the increase in the concentration of Xa gum in the formulation and its positive effect on the viscosity (increased intermolecular interactions with LBG).

#### 2.5.2. Viscoelastic Properties

The strain sweep test was performed at a frequency of 1 Hz and a strain rate of 0.01–100% to determine the linear viscoelastic region. The results of the frequency sweep (Figure 5b) at a frequency of 0.01–100 Hz below the viscoelastic region show that the changes in the storage modulus (G’) and the loss modulus (G’’) are a function of frequency. This behavior is related to the viscoelastic properties of weak gels, which are classified between real gels with covalent crosslinks and concentrated suspensions with a complex network [26]. According to the results presented by Patole et al. (2022), the measurement of viscoelastic parameters in the linear viscoelastic region leads to a reduction in structural changes during dynamic tests [51].

The higher storage modulus than the loss modulus (G’ > G”) indicates the predominance of solid viscoelastic properties in all samples; similar results for ketchup at 0.01–100 Hz [2] and 0.05–66 Hz [8] have also been reported. Changes in the storage modulus of the control sample in the frequency range of 0.01 to 100 Hz were different from other samples, which probably indicates less stability of the samples in the pumping state, while the other two samples behaved similarly to each other. In addition, the high storage modulus at high and low frequencies of the k21 and k32 samples compared to the control samples indicates a stronger structure in the stagnant (storage) state of these two samples. The results of Renou et al. (2013) on Xa gum, LBG, and their mixtures also confirm the result that if the mixed gums are used, the storage and loss modulus is 2 to 7 times higher than the modulus of each gum alone [16,19].

Changes in the loss tangent (tan δ = G’’/G’) also reflect the elastic properties of the material. At high frequencies, the K21 sample has the lowest amount of the loss tangent due to a sudden increase in the amount of storage modulus (larger slope). Similarly, Tipvarakarnkoon and Senge (2008) showed that Xa gum and LBG have a loss tangent of less than 1, which indicates the gelling phenomenon in the mixture of these gums [52]. Lorenzo et al. (2008) believe that due to the physical bonding between the polymer chains and the synergistic interactions between the hydrocolloids, a three-dimensional gel lattice forms and contributes to the development of lattice crosslinking [53]. The dual composition of specific polysaccharides exhibits unpredictable synergistic interactions. It has been well established that the synergistic process of gel formation occurs through direct bonding between two polymers and not through thermodynamic incompatibility [33]. When Xa and LBG are mixed, a network is formed whose strength depends on the preparation temperature and the weight ratio of the two gums [54]. The results indicate that the presence of acetyl groups on the Xa chain as well as the amount of galactomannan side chains play a vital role in gel formation, and increasing the number of galactose units prevents gel formation [50].

Based on the results, it was determined that no intersection between G’ and G’’ occurred at different frequencies; in other words, no crossover phenomenon was observed, which indicates physical stability at various stresses.

Using the linear regression obtained from the samples, the trend line equation of log G’ to log ω was obtained. The line slope of all three samples was in the range of 0.0197–0.218 Pa·Hz (Table 6). The lower slope indicates the dense and cohesive gel structure in the samples, which is reinforced at lower slope values (k21 and k32). Sharoba et al. (2005) reported the slope of eight commercial ketchup in the range of 0.1454–0.1032 Pa/Rad·s [2]. Further, the vertical intercept (*y*-intercept) of the studied samples was in the range of 2.3641–3.7563 Pa·s. The high values of this parameter indicate a greater sample consistency in the stagnant state, among which the k21 sample has the highest consistency.

The comparison of the complex viscosity (η*) of the samples in the linear viscoelastic range and the constant shear viscosity (η) of the flow behavior test shows that η* > η (Figure 5c) confirms the weak gel structure of the samples [22,55]. As shown in Figure 5c, at low frequencies, the amount of deformation is slower than forming new bonds, so the viscosity is constant. At high frequencies, due to the applied shear force, the weakly developed bonds are increasingly broken, which indicates the pseudoplastic flow behavior of the samples. It is also noted at high frequencies that no further structural destruction occurs, and constant viscosity and Newtonian behavior reappear. In addition, these highly regular gel networks, which hold molecules firmly in their structure, cause the gum solution to exhibit the viscoelastic properties of a weak gel [7].

### 2.6. Particle Size Distribution

Table 7 shows the results of the particle size distribution. Based on the results, it was determined that increasing the concentration of Xa gum from 32.094 to 75.06% increased the particle size and span number.

**Figure 6:**
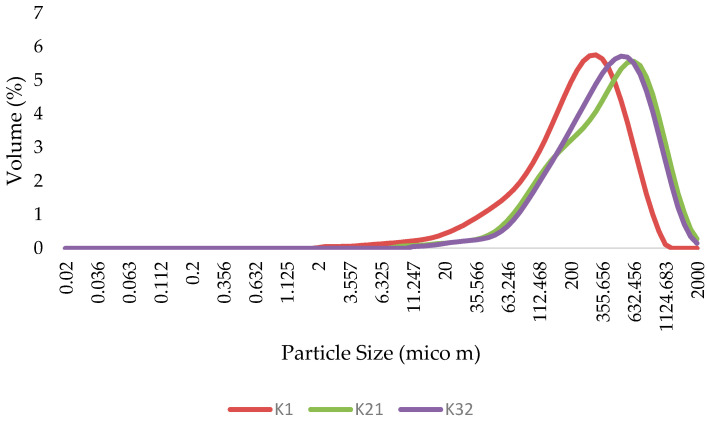
Particle size distribution of ketchups (commercial and sliceable) (K_1_ = 100% xanthan and 0% LBG, K_21_ = 24.939% LBG and 75.061% xanthan, K_32_ = 67.906% LBG and 32.094% xanthan).

Figure 6 shows the particle size distribution of various ketchup samples. According to the results, it can be seen that in all three samples, monodispersity is evident in the particles. The results also showed that for samples K21 and K32, using a mixture of LBG and Xa gum in high percentages compared to the commercial sample led to the production of products with a larger size distribution. Accordingly, as shown in Figure 6, sample K21 has a larger distribution than sample K32. In fact, the larger contact surface area between finer droplets may cause higher frictional forces opposing the free flow of the suspension upon shearing, which would increase the final viscosity and stability results presented previously [24]. Indeed, in the samples containing KM, Xa, and LBG, along with gel formation, the established network gel causes the trapping of the aqueous phase restricting serum separation and, consequently, leading to greater physical stability.

### 2.7. Microscopic Structure

**Figure 7:**
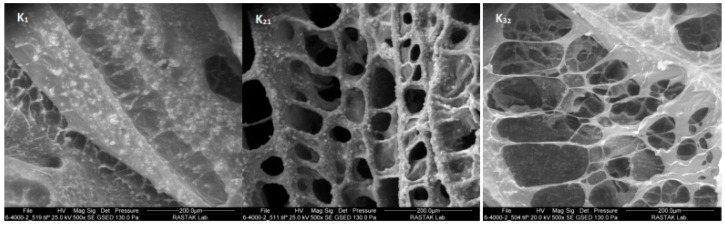
Illustration of ketchups (commercial and sliceable) by ESEM. (K_1_ = 100% xanthan and 0% LBG, K_21_ = 24.939% LBG and 75.061% xanthan, K_32_ = 67.906% LBG and 32.094% xanthan).

The results obtained from the SEM images confirmed other results in terms of the particle size distribution (Figure 7). As shown in Figure 7, the K21 sample has a more cohesive structure with stronger crosslinks, and the two K1 and K32 samples had a more irregular form with weaker crosslinks (Figure 7). This is due to the ratio of Xa to LBG in the K21 sample, which results in maximum crosslinking between the gums. The imaging results also confirm the results obtained from the viscoelastic properties and flow behaviors of the samples.

## 3. Conclusions

The examination of all the characteristics of ketchup samples containing Xa, gellan, LBG, and KM gums showed that the combined use of LBG and Xa gum creates ketchup with desirable properties in terms of texture, syneresis, and viscosity parameters for use in a variety of meat products as a filler. The use of these two gums in a 1:3 ratio (Xa:LBG) gives the best results in terms of physicochemical, textural, and rheological properties of the final sauce that can be used in the filler formulation of various meat products such as sausages.

## 4. Materials and Methods

Xanthan and gellan gum from Fisino (Hangzhou, China), konjac mannan from Everhealth (Guangzhou, China), and locust bean gum from Danisco (Copenhagen, Denmark) were obtained. All other ingredients used to prepare ketchup samples such as tomato paste (Brix = 36), vinegar, sugar, salt, and modified potato starch ADAMYL 2075 were supplied by KMC (Brande, Denmark), and spices were supplied by the R&D department of Behrouz Food Industries Co. (Tehran, Iran).

### 4.1. Sample Preparation

Ketchup samples were designed using mixture design (Design-Expert 11.0.0 software, Stat-Ease, Inc., Minneapolis, MN, USA), according to Table 8. Tomato paste, vinegar, and water were mixed with 35%, 6%, and 38.74% *w*/*w* ratios, respectively. Gellan, Xa, KM, and LBG (in total: 1.5% *w*/*w*) and other powdered ingredients (i.e., sugar: 15% *w*/*w*, salt: 2% *w*/*w*, modified potato starch: 1% *w*/*w*, and spices: 0.75% *w*/*w*) were added to the primary mixture. The mixing/homogenizing and pasteurization procedures were conducted through a vacuum mixer–homogenizer (VMH-Lab, Arkan Felez, Qazvin, Iran) for 6 min at 90 °C. Glass containers (200 mL) were filled with the ketchup samples (at 85 °C). 

### 4.2. Viscosity

The viscosity of all samples was measured using a Brookfield rotary viscometer (DV3T Rheometer, Brookfield, Middleboro, MA, USA) with a disc spindle (Spindle-RV-06) at 100 RPM and 80 °C one week after sample preparation [5].

### 4.3. Physical Stability

The method proposed by Şahin and Özdemir (2007) was used with some modifications to perform the physical stability test [5]. A 10 g sample of each ketchup was weighed in 15 mL falcons and placed in a water bath to reach a temperature of 80 °C (sausage cooking temperature). Then, by centrifugation (Universal 320R, Hettich, Tuttlingen, Germany), with a speed of 2756 g (4800 rpm), at a temperature of 40 °C for 15 min, the amount of syneresis was determined. Finally, by measuring the supernatant, the sample stability was calculated using Equation (1) [30]. This test was performed one week after sample preparation.

Physical stability = [(supernatant weight − total weight sample)/(total weight sample)] × 100(1)

### 4.4. Colorimetric Analysis

The color parameters (L, a, b) of the samples were determined using Hunterbell (Color flex, Ruston, LA, USA) after one week [30].

### 4.5. Texture Analysis

The parameters of firmness, cohesiveness, gumminess, adhesiveness, springiness, and chewiness were used to measure the texture properties of ketchup using a texture analyzer (TA-XT2 Texture Analyzer, Exponent Lite, Brookfield, USA) with a load cell of 4500 g. The samples were placed in cylindrical containers with a diameter of 26 mm and a height of 50 mm, and a cylindrical-type probe was selected (TA3-1000; D = 1.5 inches), the penetration rate of the probe into the sample was 1 mm/s and its penetration depth was 30 mm [22].

### 4.6. Viscoelastic Properties

This test was performed on commercial ketchup samples and selected samples obtained from the texture analysis, color, physical stability, and viscosity results using Design-Expert software. According to this test, the evaluation of flow behavior (steady flow behavior) and oscillating tests, including strain sweep (amplitude sweep) and frequency sweep, were performed using a rheometer with two parallel plates with a diameter of 25 mm and a plate spacing of 1 mm. In the test, the flow behavior was obtained in 0.001 to 100 1/s, and to investigate the flow properties, the following mathematical model (Equation (2)) was used and its parameters were determined:

(2) $$\frac{\eta -\eta \infty }{\eta_{0}-\eta \infty }=(1+(\lambda \gamma {)}^{2})\;\frac{n-1}{2}$$

where *η* is the apparent viscosity (Pa·s), *η*_0_ is the shear viscosity at zero shear rates (Pa·s), *η∞* is the shear viscosity at the infinite shear rate (Pa·s), λ is the release time (s), *n* is the flow behavior index (-), and γ is the shear velocity (1/s).

To evaluate the viscoelastic properties, a frequency of 1 Hz and a strain rate of 0.01 to 100% were used in the strain sweep test to determine the linear viscoelastic region. In the frequency sweep test, which is performed below the linear viscoelastic region, the amount of strain below the linear viscoelastic region was determined using a frequency of 0.01 to 100 Hz [26]. From this test, the factors of storage modulus (G’), loss modulus (G″), and complex viscosity (η^+^) were extracted as a function of frequency.

### 4.7. Particle Size Distribution Test

The Mastersizer 2000 (Malvern Instrument Ltd, Malvern, UK) equipped with a quartz cell and a laser beam with a wavelength of 634 nm at 25 °C, was used to measure particle size distribution (wet dispersion, feed rate: 50 cc/10 s, speed: 1800 mL air/min, pressure: 4 bar, mixing rate: 10 gr ketchup +30 gr reverse osmosis water, ultrasound: on). The mean surface–weight diameter (D_3,2_) (Equation (3)) and mean volume–weight diameter (D_4,3_) (Equation (4)) were obtained for selected samples and commercial ketchup samples [56].

(3) $$D_{3,2}=\sum n_{i}d_{i}^{3}/\sum n_{i}d_{i}^{2}$$

(4) $$D_{4,3}=\sum n_{i}d_{i}^{4}/\sum n_{i}d_{i}^{3}$$

### 4.8. Microstructure

In this test, a scanning electron microscope equipped with an ESEM platform (FEI ESEM QUANTA 200, Hillsboro, OR, USA) was used to study the microstructure of the samples with a device voltage of 20 kV, a pressure of 130 Pa, and a magnification of 500×.

### 4.9. Statistical Analysis

In this study, to investigate the relationship between the independent variables (Xa, KM, gellan, and LBG) and the response variables, the response surface methodology (RSM) was used. The data obtained from the design matrix tests were analyzed using Design-Expert version 11. For this purpose, from the appropriate equations, to show the relationship between each of the dependent variables in the regression model with independent variables, their mixed contour diagrams were drawn by the software. To fit the given models, the values of the R^2^ model coefficient were determined [57].

Additionally, the experiments were performed in a completely randomized design in triplicate. The results of experiments were analyzed to evaluate the significant differences between the data using one-way ANOVA using MINITAB 18 software, and to compare the mean of treatments from Tukey multiple pairwise tests, a 5% probability level (*p* < 0.05) was used.

## Figures and Tables

**Table 1:** The effect of independent variables (gellan, Xa, KM, and LBG) on the viscosity of ketchup.

Samples	Viscosity (mPa·s)
K_1_	5850 ± 14.1 ^f^
K_2_	6150 ± 10.61 ^c^
K_3_	5580 ± 1.41 ^h^
K_4_	6090 ± 0.71 ^d^
K_5_	3620 ± 7.78 ^q^
K_6_	4220 ± 4.95 ^o^
K_7_	4720 ± 3.54 ^l^
K_8_	5750 ± 3.54 ^g^
K_9_	4830 ± 2.12 ^k^
K_10_	4360 ± 14.1 ^m^
K_11_	4010 ± 0.71 ^p^
K_12_	4850 ± 7.07 ^k^
K_13_	6450 ± 2.83 ^a^
K_14_	6270 ± 5.66 ^b^
K_15_	4300 ± 0.71 ^n^
K_16_	5330 ± 0.71 ^j^
K_17_	5360 ± 9.19 ^ij^
K_18_	5940 ± 14.1 ^e^
K_19_	5390 ± 2.12 ^i^
K_20_	4360 ± 12.02 ^m^

Data were displayed in means ± standard deviation. Values with different letters were statistically significant (*p* < 0.05).

**Table 2:** The effect of independent variables (gellan, Xa, KM, and LBG) on the syneresis of ketchup.

Samples	Syneresis (%)
K_1_	3.59 ± 0.1131 ^i^
K_2_	1.348 ± 0.0297 ^kl^
K_3_	1.491 ± 0.0085 ^k^
K_4_	1.445 ± 0.0226 ^k^
K_5_	2.843 ± 0.0226 ^j^
K_6_	0.699 ± 0.0297 ^p^
K_7_	0.997 ± 0.0113 ^no^
K_8_	22.022 ± 0.0170 ^b^
K_9_	17.532 ± 0.0071 ^d^
K_10_	23.6 ± 0.0849 ^a^
K_11_	12.007 ± 0.0113 ^g^
K_12_	4.139 ± 0.0184 ^h^
K_13_	1.396 ± 0.0156 ^k^
K_14_	0.6979 ± 0.0161 ^p^
K_15_	1.144 ± 0.0127 ^mn^
K_16_	0.8487 ± 0.0137 ^op^
K_17_	21.265 ± 0.0127 ^c^
K_18_	16.758 ± 0.0127 ^e^
K_19_	1.197± 0.0156 ^m^
K_20_	12.35 ± 0.0566 ^f^

Data were displayed in means ± standard deviation. Values with different letters were statistically significant (*p* < 0.05).

**Table 3:** The effect of independent variables (gellan, Xa, KM, and LBG) on the color properties of ketchup.

Samples	L*	a*	b*
K_1_	25.38 ± 0.0283 ^efg^	31.2 ± 0.0778 ^bcde^	26.07 ± 0.0141 ^cde^
K_2_	25.95 ± 0.240 ^bc^	29.52 ± 0.0495 ^h^	24.18 ± 0.0990 ^j^
K_3_	24.52 ± 0.0495 ^j^	29.76 ± 0.1410 ^gh^	25.32 ± 0.1131 ^gh^
K_4_	24.08 ± 0.0919 ^k^	29.26 ± 0.0849 ^hi^	23.15 ± 0.1560 ^l^
K_5_	24.00 ± 0.0283 ^k^	29.44 ± 0.0707 ^h^	27.72 ± 0.0990 ^a^
K_6_	25.18 ± 0.0141 ^fgh^	28.64 ± 0.1980 ^ij^	26.8 ± 0.1560 ^b^
K_7_	23.94 ± 0.0212 ^k^	28.09 ± 0.1980 ^j^	25.99 ± 0.2120 ^cdef^
K_8_	25.13 ± 0.0354 ^gh^	32.02 ± 0.1410 ^a^	26.43 ± 0.1560 ^bc^
K_9_	25.6 ± 0.0283 ^de^	31.36 ± 0.1700 ^bcd^	25.59 ± 0.1700 ^defgh^
K_10_	25.84 ± 0.0424 ^cd^	31.66 ± 0.2970 ^ab^	26.03 ± 0.0283 ^cdef^
K_11_	25.12 ± 0.0000 ^gh^	31.44 ± 0.1840 ^abc^	25.75 ± 0.0566 ^defg^
K_12_	25.44 ± 0.0283 ^ef^	31.4 ± 0.0424 ^abcd^	25.51 ± 0.0566 ^efgh^
K_13_	25.03 ± 0.0283 ^hi^	30.83 ± 0.0990 ^cdef^	24.5 ± 0.0566 ^ij^
K_14_	27.57 ± 0.0778 ^a^	31.56 ± 0.2400 ^ab^	26.13 ± 0.1273 ^cd^
K_15_	24.61 ± 0.0849 ^j^	30.77 ± 0.3680 ^def^	25.46 ± 0.0849 ^fgh^
K_16_	26.2 ± 0.0919 ^b^	30.51 ± 0.1131 ^f^	23.5 ± 0.0141 ^kl^
K_17_	24.81 ± 0.0247 ^ij^	30.19 ± 0.0141 ^fg^	24.19 ± 0.0990 ^j^
K_18_	25.12 ± 0.0424 ^gh^	30.7 ± 0.0283 ^ef^	24.06 ± 0.0849 ^jk^
K_19_	25.37 ± 0.0566 ^efg^	30.84 ± 0.1131 ^cdef^	25.07 ± 0.1980 ^hi^
K_20_	24.72 ± 0.0424 ^j^	31.7 ± 0.0849 ^ab^	25.29 ± 0.3680 ^gh^

Data were displayed in means ± standard deviation. Values in each column with different letters were statistically significant (*p* < 0.05).

**Table 4:** The effect of independent variables (gellan, Xa, KM, and LBG) on the texture properties of ketchup.

Samples	Hardness (N)	Adhesiveness (mJ)	Cohesiveness (--)	Springiness (mm)	Gumminess (N)	Chewiness (mJ)
K_1_	4.685 ± 0.0156 ^de^	5.79 ± 0.2120 ^a^	0.6 ± 0.0707 ^abcd^	3.43 ± 0.0283 ^a^	2.77 ± 0.1700 ^d^	9.695 ± 0.0622 ^d^
K_2_	6.139 ± 0.0198 ^c^	2.48 ± 0.1131 ^ij^	0.565 ± 0.0198 ^abcd^	3.1 ± 0.2120 ^abc^	2.68 ± 0.0849 ^d^	7.86 ± 0.1700 ^e^
K_3_	8.89 ± 0.1840 ^a^	2.535 ± 0.0156 ^i^	0.535 ± 0.0184 ^bcd^	3.19 ± 0.1840 ^ab^	4.656 ± 0.1500 ^a^	14.92 ± 0.0424 ^a^
K_4_	3.786 ± 0.0255 ^e^	2.735 ± 0.1510 ^hi^	0.49 ± 0.0707 ^cd^	2.78 ± 0.1560 ^abcd^	1.809 ± 0.0198 ^e^	5.035 ± 0.1061 ^g^
K_5_	2.118 ± 0.0127 ^f^	3.02 ± 0.1273 ^gh^	0.655 ± 0.0297 ^abc^	3.105 ± 0.0071 ^abc^	1.382 ± 0.0085 ^f^	4.315 ± 0.1358 ^h^
K_6_	1.316 ± 0.0113 ^f^	2.1 ± 0.0424 ^j^	0.655 ± 0.0099 ^abc^	2.83 ± 0.0990 ^abcd^	0.864 ± 0.0042 ^g^	2.44 ± 0.0424 ^ij^
K_7_	6.274 ± 0.0042 ^c^	3.3 ± 0.1700 ^fg^	0.54 ± 0.0566 ^abcd^	3.05 ± 0.0566 ^abc^	3.358 ± 0.0156 ^c^	10.26 ± 0.0566 ^c^
K_8_	1.267 ± 0.0184 ^f^	3.355 ± 0.0170 ^efg^	0.66 ± 0.0707 ^abc^	2.66 ± 0.0283 ^bcd^	0.846 ± 0.0071 ^g^	2.34 ± 0.0990 ^ijk^
K_9_	1.086 ± 0.0354 ^f^	3.75 ± 0.1273 ^de^	0.725 ± 0.0085 ^ab^	2.77 ± 0.0990 ^abcd^	0.7925 ± 0.0049 ^g^	2.275 ± 0.0156 ^jk^
K_10_	1.431 ± 0.0042 ^f^	3.425 ± 0.0170 ^efg^	0.555 ± 0.0297 ^abcd^	2.365 ± 0.0099 ^cde^	0.796 ± 0.0028 ^g^	2.05 ± 0.0566 ^k^
K_11_	1.184 ± 0.0113 ^f^	3.385 ± 0.0325 ^efg^	0.71 ± 0.0283 ^ab^	2.735 ± 0.5150 ^abcd^	0.839 ± 0.0028 ^g^	2.31 ± 0.0141 ^jk^
K_12_	1.209 ± 0.0127 ^f^	3.67 ± 0.1131 ^def^	0.74 ± 0.0849 ^a^	2.9 ± 0.0707 ^abcd^	0.899 ± 0.0170 ^g^	2.65 ± 0.0990 ^i^
K_13_	7.644 ± 0.0453 ^ab^	3.37 ± 0.0283 ^efg^	0.555 ± 0.0325 ^abcd^	2.99 ± 0.0849 ^abcd^	4.174 ± 0.0099 ^b^	12.53 ± 0.0990 ^b^
K_14_	1.608 ± 0.0141 ^f^	2.565 ± 0.0170 ^i^	0.57 ± 0.0990 ^abcd^	2.46 ± 0.0707 ^bcde^	0.922 ± 0.0071 ^g^	2.305 ± 0.01131 ^jk^
K_15_	5.464 ± 1.4200 ^cd^	3.255 ± 0.0382 ^g^	0.48 ± 0.0566 ^cd^	2.82 ± 0.0566 ^abcd^	2.6075 ± 0.0530 ^d^	7.355 ± 0.01131 ^f^
K_16_	1.87 ± 0.0849 ^cd^	4.205 ± 0.1216 ^bc^	0.415 ± 0.0297 ^d^	1.91 ± 0.0424 ^e^	0.787 ± 0.0311 ^g^	1.545 ± 0.0269 ^l^
K_17_	1.25 ± 0.0566 ^f^	3.67 ± 0.0849 ^def^	0.555 ± 0.0085 ^abcd^	2.25 ± 0.3390 ^de^	0.694 ± 0.0170 ^gh^	1.59 ± 0.1700 ^l^
K_18_	1.596 ± 0.0042 ^f^	4.03 ± 0.0566 ^cd^	0.535 ± 0.0071 ^bcd^	2.29 ± 0.3680 ^de^	0.864 ± 0.0156 ^g^	2.15 ± 0.0566 ^jk^
K_19_	6.435 ± 0.0127 ^bc^	4.59 ± 0.1131 ^b^	0.44 ± 0.0424 ^d^	2.76 ± 0.1131 ^abcd^	2.828 ± 0.0424 ^d^	7.835 ± 0.0099 ^e^
K_20_	1.275 ± 0.0085 ^f^	4.015 ± 0.0778 ^cd^	0.43 ± 0.0707 ^d^	1.76 ± 0.0283 ^e^	0.549 ± 0.0170 ^h^	1.025 ± 0.0141 ^m^

Data were displayed in means ± standard deviation. Values in each column with different letters were statistically significant (*p* < 0.05).

**Table 5:** Carreau model parameters for filling ketchup.

Samples	η_0_ (Pa·s)	η∞ (Pa·s)	λ (s)	n (-)	R^2^	MSE	RMSE	MAPE
K_1_	5158	0.411	152.08	0.45	0.99	0.1647	0.4058	0.0161
K_21_	52213	2.454	128.88	0.54	0.99	0.3025	0.5500	0.0020
K_32_	36652	1.562	108.68	0.51	0.99	0.1243	0.3526	0.0016

K_1_ = 100% xanthan and 0% LBG, K_21_ = 24.939% LBG and 75.061% xanthan, K_32_ = 67.906% LBG and 32.094% xanthan.

**Table 6:** Slope and intercept parameters for filling ketchup.

Samples	Slope (Pa·Hz)	Intercept (Pa·s)
K_1_	0.418 ± 0.0014 ^a^	2.3641 ± 0.0002 ^c^
K_21_	0.197 ± 0.0021 ^c^	3.7563 ± 0.0002 ^a^
K_32_	0.218 ± 0.0014 ^b^	3.4943 ± 0.0000 ^b^

K_1_ = 100% xanthan and 0% LBG, K_21_ = 24.939% LBG and 75.061% xanthan, K_32_ = 67.906% LBG and 32.094% xanthan. Data were displayed in means ± standard deviation. Values in each column with different letters were statistically significant (*p* < 0.05).

**Table 7:** Particle size distribution parameters of filling ketchup.

Samples	Xanthan (%)	LBG (%)	d [3,2] (um)	Span	d [4,3] (um)
K_1_	100	0	89.539 ± 0.0099 ^c^	2.191 ± 0.0007 ^b^	264.258 ± 0.004 ^c^
K_21_	75.061	24.939	199.419 ± 0.0060 ^b^	2.214 ± 0.0049 ^a^	474.560 ± 0.006 ^a^
K_32_	32.094	67.906	215.095 ± 0.0010 ^a^	2.124 ± 0.0042 ^c^	448.923 ± 0.016 ^b^

K_1_ = 100% xanthan and 0% LBG, K_21_ = 24.939% LBG and 75.061% xanthan, K_32_ = 67.906% LBG and 32.094% xanthan. Data were displayed in means ± standard deviation. Values in each column with different letters were statistically significant (*p* < 0.05).

**Table 8:** Experimental matrix based on the independent variables of the mixture design formula in (%).

Treatments	Konjac Mannan (%)	Locust Bean (%)	Gellan (%)	Xanthan (%)
K_1_	0 (Level 1)	50 (Level 4)	50 (Level 4)	0 (Level 1)
K_2_	50 (Level 4)	0 (Level 1)	0 (Level 1)	50 (Level 4)
K_3_	12.5 (Level 2)	62.5 (Level 5)	12.5 (Level 2)	12.5 (Level 2)
K_4_	62.5 (Level 5)	12.5 (Level 2)	12.5 (Level 2)	12.5 (Level 2)
K_5_	12.5 (Level 2)	12.5 (Level 2)	62.5 (Level 5)	12.5 (Level 2)
K_6_	0 (Level 1)	0 (Level 1)	50 (Level 4)	50 (Level 4)
K_7_	0 (Level 1)	50 (Level 4)	0 (Level 1)	50 (Level 4)
K_8_	50 (Level 4)	0 (Level 1)	50 (Level 4)	0 (Level 1)
K_9_	50 (Level 4)	50 (Level 4)	0 (Level 1)	0 (Level 1)
K_10_	50 (Level 4)	50 (Level 4)	0 (Level 1)	0 (Level 1)
K_11_	0 (Level 1)	50 (Level 4)	50 (Level 4)	0 (Level 1)
K_12_	0 (Level 1)	100 (Level 6)	0 (Level 1)	0 (Level 1)
K_13_	12.5 (Level 2)	12.5 (Level 2)	12.5 (Level 2)	62.5 (Level 5)
K_14_	0 (Level 1)	0 (Level 1)	0 (Level 1)	100 (Level 6)
K_15_	25 (Level 3)	25 (Level 3)	25 (Level 3)	25 (Level 3)
K_16_	50 (Level 4)	0 (Level 1)	0 (Level 1)	50 (Level 4)
K_17_	50 (Level 4)	0 (Level 1)	50 (Level 4)	0 (Level 1)
K_18_	100 (Level 6)	0 (Level 1)	0 (Level 1)	0 (Level 1)
K_19_	0 (Level 1)	50 (Level 4)	0 (Level 1)	50 (Level 4)
K_20_	0 (Level 1)	0 (Level 1)	100 (Level 6)	0 (Level 1)

## Data Availability

All data are presented in the manuscript.
